# Cognitive Correlates of Functional Disruption at Psychosis Onset: Unique Relevance of Visual Cognition

**DOI:** 10.3390/jcm14103308

**Published:** 2025-05-09

**Authors:** Alessia Avila, Ricardo Coentre, Tiago Mendes, Pedro Levy, Matteo Cella, Filipa Novais

**Affiliations:** 1 University Clinic of Psychiatry and Medical Psychology, Faculty of Medicine, University of Lisbon, 1649-028 Lisbon, Portugal; ricardo.coentre@ulssm.min-saude.pt (R.C.); tiagohmendes@outlook.pt (T.M.); fnovais@campus.ul.pt (F.N.); 2 Neuroscience and Mental Health Department, North Lisbon University Hospital (CHULN), 1649-028 Lisbon, Portugal; pedro.levy@chln.min-saude.pt; 3 Department of Psychology, Institute of Psychiatry Psychology and Neuroscience, King’s College London WC2R 2LS, UK; matteo.cella@kcl.ac.uk

**Keywords:** early phase psychosis, visual cognition, negative symptoms, path analysis

## Abstract

**Background**: Cognitive impairment is a common feature of schizophrenia spectrum disorders and has been associated with functional disruption preceding the onset of psychosis. Understanding how cognitive deficits interact with clinical symptoms and functioning in early psychosis remains challenging. In this study, we aim to investigate whether a distinct “cognitive signature” characterizes functional disruption at the onset of psychosis. **Material and Methods**: Clinical, cognitive, and functional data were collected from 101 first episode psychosis patients at their first hospitalization. Stepwise regression models were used to identify predictors of global functioning and symptom severity at the time of onset, as well as diagnostic outcomes at discharge. Path analysis was used to explore the relationship among symptom severity, cognition, and functional outcomes. **Results**: Deficits in visual memory were selectively predictive of lower functioning and higher global symptom severity at the time of psychosis onset. Reduced visual-spatial abilities were also associated with unemployment at the time preceding hospitalization and predicted a non-affective schizophrenia spectrum diagnosis at discharge. Path analysis found that visual memory fully mediated the relationship between negative symptoms and level of functioning. **Conclusions**: Impairment in visual cognition seems to be uniquely associated with functional impairment and global symptom severity at the onset of psychosis and to mediate the relationship between negative symptoms and functioning. The results might indicate a primary relevance of visual cognitive aspects in marking functional disruption and symptom exacerbation at psychosis onset. This might have implications for early detection and inform treatment plans.

## 1. Introduction

Cognitive deficits represent a key symptomatic domain in schizophrenia spectrum disorders, which emerge in prodromal phases and persist throughout all stages of the illness development [1,2]. Impairments affect multiple cognitive domains and have been consistently associated with functional loss [3]. A direct relationship between cognitive performance and real-world functioning has been well established in schizophrenia spectrum disorders [4] and confirmed in first episode psychosis (FEP) cohorts [5]. In particular, cognitive impairment has been linked to functional disruption preceding full blown psychosis onset, leading to higher rates of hospitalizations and longer inpatient stay [6]. Despite the recent advances in data analysis, which allowed for a greater understanding of the role cognitive symptoms hold in contributing to functional impairment in psychosis [7,8], the interplay between clinical and cognitive symptoms and their mutual impact on functional outcomes remain challenging to disentangle. Various factors might contribute to this issue, particularly the high variability of cognitive profiles in patients with psychosis, and the inherent difficulty in reducing sample heterogeneity in clinical studies on this population. Efforts to reduce heterogeneity have included focusing on specific phases of the illness and trying to identify clinical and cognitive subtypes, for instance, via clustering methods [9,10,11,12,13,14,15]. Several studies on first episode psychosis patients have validated cognitive subtypes by showing a significantly lower baseline functioning and worse functional outcomes in cognitively impaired patients [9,16,17,18,19]. Studies have also shown how cognitive sub-groups differ in level of negative symptoms [10,20,21] and in measures of brain volume [11,16,22]. In an attempt to further elucidate the associations among cognitive, functional, and clinical aspects in the very early phase of psychosis emergence, we set out to answer the question of whether is possible to identify a “cognitive signature” of functional disruption at psychosis onset. Moreover, we intend to clarify whether specific cognitive deficits relate to symptom severity and diagnostic outcomes, at this stage. This could help elucidate cognitive contributors to functional decline at psychosis onset and might be relevant for identifying cognitive correlates directly linked to etiologic mechanisms.

## 2. Materials and Methods

### 2.1. Participants

Patients were recruited from the mental health department of a public hospital in Lisbon (Portugal) and assessment was conducted as part of a standard protocol based on those internationally adopted [23,24]. Participants were included in the study if they were aged between 18 and 35, hospitalized for the first time for a psychotic episode, antipsychotic naïve, clinically stable, and had received a discharge notice. The exclusion criteria were as follows: previous hospitalization for a psychiatric disorder, previous exposure to antipsychotic learning disability (IQ < 70), or presenting with neurological co-morbidities affecting cognition.

### 2.2. Procedures

Patients subsequently admitted by the First Episode inpatient team between 2018 and 2021 were screened for inclusion criteria. Once patients reached clinical stability within a definite treatment protocol (i.e., positive symptoms remission following [25], they received a discharge plan. Over the two days before discharge, patients underwent a full clinical and neuropsychological assessment and were asked to sign an informed consent to use their data for research purposes. Data on clinical and functional assessments were always collected by integrating information from patients and carers to ensure reliability and minimize recall biases. The assessment of global functioning was mostly based on information provided by carers. Patients were then discharged on a formal diagnosis and given an outpatient appointment. The study was conducted in accordance with the Declaration of Helsinki and approved by the Hospital Ethical Committee (ID 62/19, 18 April 2019) and by the Faculty of Medicine’s Scientific Committee.

### 2.3. Measures

*Socio-demographic characteristics*

Multiple socio-demographic characteristics were collected and are reported in Table 1. Information about previous substance use and age at first use were collected. Antipsychotic medication dosage at the time of assessment was registered and dosage transformed into Chlorpromazine equivalents; the Defined Daily Doses (DDD) method was used, with values rounded to the nearest 25 mg increment [26].

#### 2.3.1. Clinical Assessment

Severity of psychotic symptoms was assessed using the Positive and Negative Syndrome Scale (PANSS) [27]. The total score on PANSS was considered an index of global symptom severity. Moreover, diagnosis at discharge was registered and the sample was classified according to whether patients were discharged on an affective (F32.3; F30.2) or non-affective psychosis diagnosis, including Schizophrenia; Brief Psychotic Disorder and Drug Induced Psychotic episode (F20.0; F23.0; F12.5). We opted for this classification in order to reduce the number of comparisons given our sample size, while maintaining a relevant clinical distinction in our diagnostic outcomes. Affective symptoms were assessed via the Montgomery and Asberg Depression scale (MADRS) [28] and the Young Mania Rating Scale (YMRS) [29].

#### 2.3.2. Neuropsychological Assessment

We administered a complete battery of widely adopted tests, validated in the Portuguese population and for which national normative values were available. In particular, sustained attention was tested via the Tulouse Pierron cancelation test; verbal memory and learning were assessed via the California verbal learning Test (CVLT) [30] and the Trail Making Test (TMT) [31], part A was used to assess speed of processing; various aspects of executive functioning were also measured: task switching was assessed via TMT, part B, and working memory via the Digit Span backward subtest; flexibility and abstraction were tested via the Wisconsin Card Sorting Test (WCST) [32], while verbal fluency was assessed via a semantic verbal fluency task (1 min) [33]. Visual memory and visuo-spatial abilities [34] were measured via the Rey–Osterrieth complex figure (ROCF) [35,36]; administration and scoring followed the original protocol [37] because normative data were not available for other scoring systems. Regarding the copy type score, we opted for not restricting the results to the first 4 types commonly found in adults, as studies have demonstrated that in psychiatric and neuropsychiatric populations, this might not be able to capture the full spectrum of possible outcomes [34,38]. Neuropsychological assessment was conducted by the same professional on all patients, with the exception of 4 subjects. As this represents less than 4% of the total sample, we opted for not performing an inter-rater reliability analysis. In order to minimize reliability biases, scoring on the ROCF was reassessed by the first rater, and putative discrepancies were solved.

#### 2.3.3. Level of Functioning

Current social functioning was measured via the Personal and Social Performance scale (PSP) [39], capturing functioning in the month prior to hospitalization. As a measure of role functioning, we also recorded employment status at the time of hospitalization, considering whether patients were involved in active employment or education.

### 2.4. Statistical Analysis

First, separate univariate linear regressions were conducted using SPSS Version 29, with demographic, clinical, and cognitive variables as predictors of the 4 outcome variables: level of functioning (score on PSP) and symptom severity (PANS total) were analyzed as continuous variables; employment status at the time of onset and diagnosis at discharge were analyzed as categorical, dichotomous variables. Variables with significant *p* values were then entered into a multivariate stepwise regression model, considering the same outcome measures. Therefore, two different stepwise multivariate models were used to identify predictors of the level of functioning (score on PSP) and symptom severity (PANS total) and two stepwise logistic regression models were built with employment status at the time of onset and diagnosis at discharge as the outcome measures. R^2^ and Odd Ratios for 95% CI were retrieved as measures of effect size. Collinearity amongst predictors was assessed via the VIF statistics. Finally, mediation analysis was conducted using ordinary least squares path analysis to test the putative mediating effect of the identified cognitive variables, in the relationship between clinical symptoms and level of functioning. Therefore, clinical symptoms were set as predictor (X), cognition variables emerging from stepwise models were considered as mediators (M), and level of functioning was considered the outcome variable (Y). Analyses were performed using the PROCESS macro for SPSS Version 29 [40]

## 3. Results

### 3.1. Sample Characteristics

The sample considered was 101 FEP patients. Participants’ average age was 24 years old (SD = 5.7), and the average level of education was 12 years (SD = 3.1), which in Portugal, represents the end of secondary school. Most patients were Caucasians (73% Portuguese and 7.5% from other EU countries), with some (19%) being form minority groups, mainly belonging to Portuguese ex-colonies (Brazil, Angola, Cape-Verde). At the time of assessment, patients had reached a stable dosage of antipsychotic (AP) medications and 97.3% (N = 98) were taking Second Generation APs, including 5.6% participants who were prescribed Clozapine (N = 5). The remaining patients (N = 3) were prescribed a First Generation AP. Average exposure to neuroleptics was 21 days. The results on clinical and cognitive characteristics are reported in Table 1. Compared to normative controls, patients displayed broad impairments in most cognitive domains, and the size of impairment is recorded as Cohen’s d from standardized z-scores. The sample was assessed under a relatively controlled environment, with no current use of psychoactive recreational substances at the time of assessment.

### 3.2. Association with Functional Impairment

In a first stepwise multivariate model, visual memory (immediate recall, ROCF) was selectively predictive of functional impairment at the time of hospitalization (ß = 0.297, *p* = 0.008, CI = 0.165, 1.067). The model also retrieved a significant effect of levels of education (ß = 0.322, *p* = 0.005 CI = 0.514, 2.799) and, understandably, medication dosage (ß = −0.263, *p* = 0.013, CI = −0.031, −0.004). Overall, the model had a medium effect size and explained just under 38% of data variance (R^2^ = 0.378). Consistently with these findings, a second stepwise logistic model showed that visual-spatial abilities were strongly associated with employment status at the time of psychosis onset (O.R. = 0.564, *p* = 0.009, CI = 0.367, 0.868) and pre-morbid adjustment also significantly affected this outcome (Exp(B) = −0.935, *p* = 0.014. CI = 0.887, 0.987).

### 3.3. Association with Symptoms Severity

In a third stepwise multivariate model, visual memory emerged again as the cognitive domain more strongly associated with global symptom severity, as measured by the PANSS total score (R^2^ = 0.264; ß = −0.214; *p* = 0.019, CI = −0.840, −0.076). Depressive symptoms and level of medication were also selected as significant predictors in the model (ß = −0.225; *p* = 0.012, CI = 0.061, 0.482; and ß = −0.339; *p* = <0.0005, CI = 0.011, 0.036, respectively).

### 3.4. Diagnostic Outcomes

In the last stepwise logistic regression model, reduced visual-spatial abilities were predictive of non-affective diagnosis at discharge (R^2^ = 0.376; O.R. = 0.498, *p* = 0.036, CI = 0.244, 1.018). Level of functioning at the time of hospitalization was also selected as predictor, although with moderate significance (O.R. = 1.063; *p* = 0.049, CI = 1.000, 1.129).

The results from the regression models are summarized in Table 2.

### 3.5. Path Analysis

**Figure 1:**
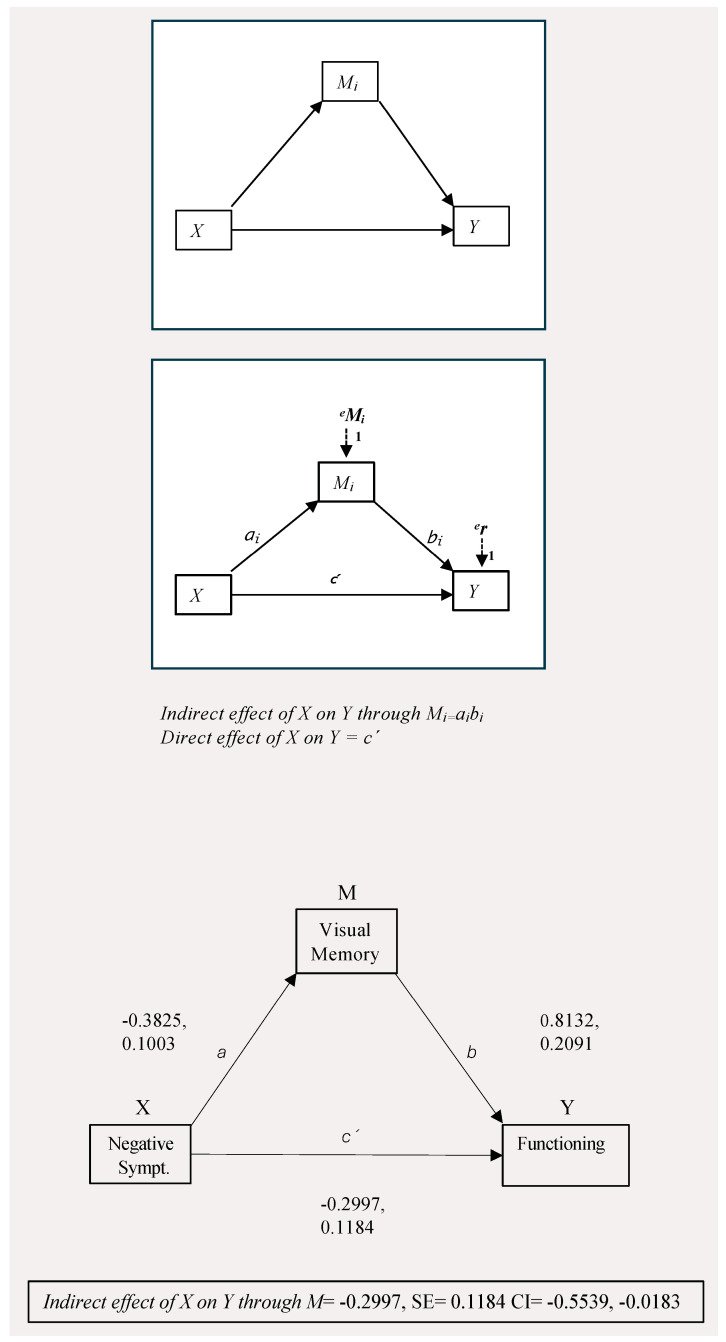
Mediation model of interaction among visual memory (M), symptom severity (X), and functioning (Y).

We further explored the putative mediating effect of cognitive variables on the relationship between clinical symptoms and functioning, via path analysis, using the PROCESS macro, model 4. Considering the results from the previous stepwise regressions, we initially tested a model including functioning as the outcome variable (Y), symptom severity (PANSS total) as predictor (X), and visual memory as mediator (M). This model only retrieved evidence for partial mediation (direct effect of X on Y, (c) = −0.2628, CI = −0.4567, −0.0689, *p* = 0.0085; indirect effect (c’) = −0.1360, CI = −0.2399, −0.0454). In a second model, we found evidence that visual memory mediated the relationship between negative symptoms and level of functioning. Bootstrapped confidence intervals for the indirect effect (a*b = −0.2997, CI = −0.5539, −0.0813) indicated that visual memory fully mediated the effect of negative symptoms on functioning in the sample considered. The results are displayed in Table 3, while graphical representations of the analyses are reported in Figure 1.

## 4. Discussion

In the present study, we investigated the relationship among cognitive, clinical, and functional variables in a cohort of first episode psychosis patients at their first hospitalization and medication exposure, with the goal of identifying a putative “cognitive signature” of functional disruption at psychosis onset. Considered together, our results seem to pinpoint a primary relevance of the visual cognition domain, encompassing visual memory and visual spatial abilities. Visual cognition can be broadly intended as the abilities underpinning the processing, storage, and recall of visual information [41] and is emerging as a particularly relevant area of cognitive disfunction in psychosis [42]. Visual–cognitive abnormalities have been well identified in chronic as well as in first episode patients [43] and lately, impairment in this domain was suggested to constitute a potential risk factor for psychosis development itself [44]. Independent metanalyses on ultra-high risk samples, in fact, have identified deficits in visual processing tasks as one of the strongest predictors of transitioning to a psychotic disorder [45,46]. In our sample, deficits on measures of visual cognition were uniquely associated with functional impairment in the very early phases of psychosis emergence, compared to the other assessed cognitive domains. Disruption in visual processing has been previously identified as a cognitive mechanism preceding functional deterioration in psychosis relapse, regardless of worsening positive symptoms [47]. Interestingly, in a recent network analysis comparing early and late phase schizophrenia patients, visual cognition and disorganization were retrieved as the more central nodes, standing at the crossway between cognitive domains, metacognition, and measures of functioning in both cohorts [48]. Visual cognition might be involved in determining how people manipulate information about themselves and others and how they relate events, being therefore a crucial requisite for meta-cognitive processing, which in turn, directly influence functioning [49]. In our findings, premorbid adjustment was also included in the final model. This is consistent with a neurodevelopmental hypothesis of psychosis and is in line with conclusions from several studies on both ultra-high risk (UHR) and first episode cohorts, which found associations between premorbid adjustment and both functional and cognitive outcomes [50,51,52]. The present study builds on previous literature, uncovering the relevance of visual cognition specifically amongst other cognitive domains: it would be of great interest to investigate whether impairment in this domain is somewhat related to aspects of functional adjustment already during development and could constitute an early predictor of adverse clinical and cognitive trajectories. An association between visual-spatial abilities and role functioning was also established in the present study, in line with previous findings in first episode populations, linking visual cognition to better job tenure and better vocational outcomes [53,54]. Visual spatial abilities would, in fact, capture aspects of executive functioning directly related to information processing and organization, which then influences functional abilities at a broader level [55]. Taken together, these results seem to sustain the hypothesis of a specific relevance of the visual cognition domain in contributing to functional disruption at psychosis onset and might help elucidate neurocognitive mechanisms relevant to psychosis pathogenesis. In fact, recent imaging studies identified a phase-specific functional connectivity disruption between the visual network (VN) and the default mode network (DMN) at psychosis emergence, which might constitute a putative neural mechanism linking visual processing disfunction and vulnerability towards psychosis [56]. Pathways between DMN and visual networks have also been linked to the ability to process sustained emotional experiences and might be crucial to social cognition [57]. Here, an association between global symptom severity and visual cognition was also detected. Impairments in visual processing have been related to overall clinical severity in patients with schizophrenia and have been associated with the emergence of positive symptoms via a disruption in fronto-subcortical pathways [58]. Findings from a recent multicentric study on FEP patients revealed an association among better visual processing, fewer negative symptoms, and better functioning [51], again pointing at a connection between this cognitive domain and functional and clinical aspects. More consistent indications of an interplay between visual cognition and clinical symptoms are emerging in the literature [59], with implications for the planning of future interventions. In our model, depressive symptoms were also significantly associated with overall psychosis symptom severity. Although depressive symptoms are commonly present in schizophrenia spectrum disorders, their relationship with psychotic symptoms remains complex. In particular, it is unclear whether their appearance precedes or is secondary to the onset of positive symptoms. In our study, we detected significant associations between depressive and psychotic symptoms in an early phase of the illness onset, suggesting that depressive symptoms might indeed precede psychosis outbreak. On the one hand, depressive symptoms might lead to detrimental behavioral changes, such as social withdrawal, isolation, and functional impairment, which might worsen the impact of emerging psychotic experiences [60]. Another possible mechanism might involve the impact of depression on cognitive abilities, which would in turn exacerbate the intensity of psychotic symptoms. Interestingly, previous findings indicated that some depressive symptoms contribute to the development of specific neurocognitive impairments, particularly aspects of executive functioning and visual memory [61]. Finally, there is a known phenomenological overlapping between depressive and negative symptoms, with meta-analytical findings confirming a small but significant relationship between the two [62]. Future research should further investigate the multidirectional link between depressive symptoms and other domains of psychosis and clarify putative etiologic overlapping. We found evidence of a mediating effect of visual memory in the relationship between negative symptoms and level of functioning. In a recent study, visual cortical alterations were associated with the severity of negative symptoms in a drug naïve first episode cohort [63], while results from a trial on cognitive remediation found that the enhancement of visual cognition predicted improvement in negative symptoms [64]. Visual cognition might be important for the perception of visual-social stimuli (facial and nonverbal expressions, for instance) and influence the ability of visualizing previously experienced rewarding stimuli [63]. The present findings pose visual cognition at a crossway between negative symptoms and functional outcomes, contributing to disentangling the complex interplay of various symptomatic domains. Future research should explore the potential neurobiological mechanism underpinning this intriguing interaction. A small study found that visual memory uniquely predicted anhedonia in a sample of FEP but not bipolar patients, implicating a specific association between these domains in non-affective psychosis [65]. Somewhat consistently, we found that patients performing more poorly on the visual-spatial task were significantly more likely to be discharged on a non-affective psychosis diagnosis. This implies that visuo-spatial cognitive difficulties might reflect underling pathogenic processes specific to non-affective psychosis [66,67,68]. Findings from the PRONIA consortium also concluded that visual impairments were specific to the onset of psychosis, rather than affective disorders [69]. More recent results from the same international group found that performance in visual memory significantly differentiated between recent onset psychosis and recent onset depression patients [70]. Future research should integrate disruptions in visual cognition within the broader conceptualization of the etiopathogenesis of non-affective psychosis and deepen the understanding of the specific neurobiological mechanism involved.

Our study has several limitations. First and foremost, the use of data from cross-sectional rather than multiple assessments greatly limits the strengths of our conclusions. A one year follow up assessment is already underway for the same cohort and the results should be published in the near future. Finally, our sample size was relatively small; bigger cohorts are deemed necessary to further elucidate the explored mechanisms, using more complex statistics such as network analysis. On the other hand, we can claim a few strengths to our design. Our sample was rather homogeneous and included patients at their very first episode of psychosis, who were never previously exposed to APs. Patients were assessed under controlled conditions and had no concurrent substance use at the time of assessment. Due to the fair homogeneity of our sample, we can assume that our results realistically captured relevant mechanisms.

## 5. Conclusions

Considered together, our results indicate that impairment in visual cognition is uniquely associated with functional impairment and global severity at illness onset and might be specific to non-affective psychosis. If confirmed in longitudinal designs, the findings could provide a valuable tool for the early identification of patients with a worse prognosis and aid treatment planning. For instance, CR interventions specifically targeting visual cognitive domains might be prioritized and contribute to both clinical and functional improvement.

## Figures and Tables

**Table 1:** Demographic and cognitive characteristics of sample.

Variables	Mean (SD)/N (%)			
*Demographics*				
Gender, male	80 (75.7)			
Age	24 (5.6)			
Ethnicity				
White Caucasian Portuguese	86 (80.5)			
	78 (73)			
Other European	8 (7.5)			
Ethnic minorities	21 (19.5)			
Years of Education	12 (3.1)			
Duration of Illness (months)	14.4 (21) ^a^			
*Chlorpromazine Equivalent DDD*	423 mg (229 mg) ^b^			
*AP’s class ^c^*				
First Generation Second Generation	3 (3.2) 98 (97.3)			
Of which Clozapine	5 (5.6)			
*Lifetime Substance use ^d^*	74 (69.1)			
Age at First Use (any substance)	15.7 (3.6)			
Cannabis	65 (60.7)			
Other substances †	28 (26.2)			
*Clinical Symptoms*				
PANSS Total ^e^	80.3 (16.1)			
PANSS Positive	24.3 (6.5)			
PANSS Negative	16.5 (7.3)			
PANSS General	39.5 (9.4)			
*SANS Total ^f^*	26.9 (15.4)			
*Diagnosis at discharge ^g^*				
Schizophrenia (F.20.0) Brief Psychotic Disorder (F23.0)/Drug Induced Psychosis (F.12.25) Depressive/Manic episode with psychotic symptoms (F.32.3; F.30.2)	45 (44.5) 35 (34.6) 20			
*Pre-morbid adjustment ^h^* PAS General PAS Infancy PAS Primary Adolescence PAS Secondary Adolescence PAS Adulthood	21.8 (11.04) 6.9 (4.6) 10.1 (5.4) 10.7 (5.9) 7.3 (4.6)			
*Current functioning ^i^*				
PSP	38.2 (15.8)			
Employment Status (Unemployed) ^j^	42 (41.6)			
**Cognitive domains and measures** ‡	**Mean(SD)/Frequencies (%)**	Cohen’s d * **FEP vs. Normative**	95%CI *	
			***Lower***	***Upper***
*Attention*				
Tulouse Pieron—Dispersion Index (%)	40 (32.5)	2.1	*0.0207*	*0.7300*
*Verbal Memory and Learning CVLT*				
Total Free Recall (A1–A5)	41.6 (9.7)	2.7	*0.1541*	*1.0084*
Total Long Delayed Free Recall (20 min.)	8 (3.2)	3.2	*0.3006*	*1.2814*
*Executive Functioning*				
TMT A (time in seconds) TMT B	42.6 (20.2) 111 (61.4)	0.06 1.2	*−0.1916* *−0.1025*	*0.2036* *0.4164*
WCST Preservatives Errors	15.2 (11.7)	0.5	*−0.1567*	*0.2623*
WCST N. correct categories	4.3 (2.1)	1.8	*−0.0297*	*0.6113*
Digit Span backwards	3.7 (1.3)	0.9	*−0.1280*	*0.3406*
Verbal Fluency (1 min)	17 (5.6)	0.9	*−0.1280*	*0.3406*
*Visual Memory and Visual Spatial Abilities*				
ROCFT—Memory recall (4 min)	15.2	1.5	*0.0704*	*0.5067*
ROCFT—Copy Type	**Category’s Percentile**			
		**Patients**	**Normative Controls**	
	10th	I	III/IV	
	25th	II	II	
	50th	III	I	
	75th	IV	I	
	95th	V	I	

DDD: Daily Defined Doses; AP’s: Antipsychotics; PANS: Positive and Negative Syndrome Scale; SANS: Scale for the Assessment of Negative Symptoms; PAS: Premorbid Assessment Scale. Missing data reported for >5 <10 cases: (^a,b^) data missing for 5 participants; (^c^) data missing for 6 participants; (^d^) data missing for 7 participants; (^e^) data are missing for 4 participants; (^f^) data missing for 7 participants; (^g^) data missing for 7 participants; (^h^) data for PAS Inf., Ado1, Ado2, Gen missing for 8 participants; (^i^) data missing for 8 participants; (^j^) data missing for 6 participants. † other psychoactive substances: cocaine, MDMA, LSD, heroin; ‡ 5 participants interrupted the execution of WCST and 3 the TP test: these data were scored as missing; 2 patients interrupted the memory task of RCFT and scored based on the completed sections. * Cohen’s *d* and CI were manually calculated based on z-scores and indicate the number of SDs below the norm.

**Table 2:** Results from stepwise regression models.

***Functional Impairment***								
**Current Social Functioning** (time of Hospitalization and Month Prior)								
**Stepwise Multivariate Regression Model**								
**Predictors**	**Effect Size**	**Unstand. B**	**S.E.**	**ß**	**t**	***p* Value**	**95% CI**	
							Lower	Upper
	^a^R^2^ = 0.378							
Visual Mem.		0.616	0.226	0.297	2.732	0.008	0.165	1.067
Educ. Level		1.656	0.571	0.322	2.900	0.005	0.514	2.799
Medic. Level		−0.017	0.007	−0.263	−2.550	0.013	−0.031	−0.004
**Employment Status** (time of hospitalization and month prior)								
**Stepwise Logistic Regression Model**								
**Predictors**	**Effect size**	**Unstand. B**	**S.E.**	**Exp(B)**	**Wald**	***p*** **Value**	**95% CI**	
	^a^R^2^ = 0.275						Lower	Upper
		−0.572	0.220	0.564	6.774	0.009	0.367	0.868
Visual-Spatial								
General Pre-morbid Adj.		−0.067	0.027	935	6.039	0.014	0.887	0.987
***Symptom Severity***								
**Stepwise Multivariate Regression Model**								
**Predictors**	**Effect size**	**Unstand. B**	**S.E.**	**ß**	**t**	***p*** **Value**	**95% CI**	
	^a^R^2^ = 0.255							
Medic. level		0.024	0.006	0.339	3.754	<0.0005	0.011	0.036
Depressive Symptoms		0.272	0.106	0.225	2.563	0.012	0.061	0.482
Visual Mem.		−0.458	0.192	−0.214	−2.380	0.019	−0.840	−0.076
***Diagnostic Outcome*** (non-affective vs. affective psychosis)								
**Stepwise Logistic Regression Model**								
**Predictors**	**Effect size**	**Unstand. B**	**S.E.**	**Exp(B)**	**Wald**	***p*** **Value**	**95% CI**	
	^a^R^2^ = 0.376						Lower	Upper
								
Current. Func.		0.061	0.031	1.063	3.831	0.049	1.000	1.129
Visual-Spatial		−0.697	0.365	0.498	3.647	0.036	244	1.018

**Table 3:** Results from mediation model.

		M (Visual Memory)						Y (Functioning)				
		ß	SE	*p*	95% CI			ß	SE	*p*	95% CI	
Antecedent					upper	lower					upper	lower
X (Negative Symptoms)	*a*	−0.3825	0.1003	0.0015	−0.5278	−0.1292	*c’*	−0.3768	0.2083	0.073	−0.7908	0.0373
M (Visual Memory)		-	-	-	-	-	*b*	0.9123	0.2091	<0.001	0.4968	10.3279
		R^2^ = 1087						R^2^ = 0.2619				
		*F*(1, 100) = 10.733 *p*0 = 0.0015						*F*(2, 98) = 15.436 *p* = <0.001				
							**Total Effect**		**SE**	***p***	**95%CI**	
							C = c’+ a*b				upper	lower
							−0.6764		0.2159	0.0023	−1.1055	−0.2475
							**Indir. Effect**		**SE**	***p***	**95%CI**	
							a*b				upper	lower
							−0.2997		0.1184	-	−0.5539	−0.0183

## Data Availability

The data presented in this article are part of an ongoing study. Please contact the first author to request access.
